# HPLC Quantification of 4-Nitrophenol and its Conjugated Metabolites from Bile

**DOI:** 10.3797/scipharm.1106-22

**Published:** 2011-08-18

**Authors:** Attila Almási, Emil Fischer, Pál Perjési

**Affiliations:** 1 Institute of Pharmaceutical Chemistry, University of Pécs, Rókus Street 2, H-7624 Pécs, Hungary; 2 Department of Pharmacology, University of Pécs, Szigeti Street 12, H-7624 Pécs, Hungary

**Keywords:** 4-Nitrophenol, Glucuronic acid conjugate, Sulfate conjugate, RP-HPLC, Intestinal metabolism

## Abstract

An isocratic ion pair RP-HPLC method with UV-Vis detection has been developed and validated for simultaneous analysis of 4-nitrophenol (PNP), 4-nitrophenyl β-glucuronide (PNP-G), and 4-nitrophenyl sulfate (PNP-S) in rat bile samples using 4-ethylphenol (ETP) as internal standard. Chromatographic separation was achieved on a C_18_ column by isocratic elution with a mobile phase consisted of methanol-0.01 M citrate buffer pH 6.2 (47:53 v/v) containing 0.03 M TBAB. The flow rate was 1.0 ml min^−1^, the detection was affected at 290 nm. Calibration plots were generated over the concentration range 1–100 μM PNP, PNP-G, PNP-S with a common lower limit of quantification of 2.5 μM. Intra- and inter-day precision and repeatability were determined at six different concentrations. Results obtained by application of the method for determination of PNP, PNP-G and PNP-S in bile fractions collected during intestinal perfusion of PNP in hyperglycemic rats are presented.

## Introduction

**Fig. 1:**
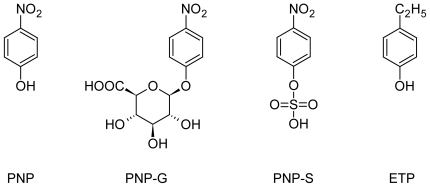
Structure of 4-nitrophenol (PNP), 4-nitrophenyl β-glucuronide (PNP-G), 4-nitrophenyl sulfate (PNP-S) and 4-ethylphenol (ETP)

After oral administration of the phenolic compounds, the hepatic and intestinal glucuronic acid and sulfate conjugations have the most important roles as metabolic transformations [1]. 4-Nitrophenol (PNP) is excreted almost exclusively as its glucuronide (PNP-G) and sulfate (PNP-S) (Fig. 1) [2, 3]. Because of its simple and well characterized metabolic profile PNP is widely used as a model substrate to evaluate the influence of diseases, nutrient deficiencies and other physiologically altered conditions on conjugative drug metabolism in animal studies both *in vitro* and *in vivo* [4–6].

Earlier we set up an *in vivo* experimental protocol for investigation of intestinal metabolism of 4-nitrophenol (PNP) in control and hyperglycemic rats [7]. In the course of our studies, an isocratic ion-pair reversed phase HPLC (RP-HPLC) assay coupled with UV detection for simultaneous determination of PNP, PNP-G and PNP-S in the intestinal perfusates has been developed [8]. As a continuation of our work in this field we have developed a modified isocratic ion-pair RP-HPLC-UV method using 4-ethylphenol (ETP) as internal standard to quantify PNP and its metabolites in the bile samples of the experimental animals. This work describes details (focusing on linearity, within-day precision and day-today precision) of the optimized easy to run reversed phase HPLC-UV assay to quantify 4-nitrophenol (PNP), 4-nitrophenyl *β*-glucuronide (PNP-G) and 4-nitrophenyl sulfate (PNP-S) in a large number of samples generated in animal experiments.

## Results and Discussion

### Results

**Fig. 2:**
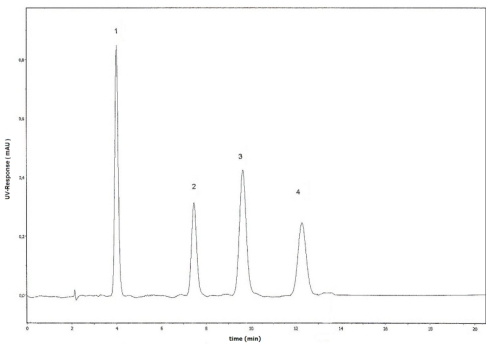
HPLC chromatogram of 4-nitrophenol (PNP) (2), 4-nitrophenyl β-glucuronide (PNP-G) (1), 4-nitrophenyl sulfate (PNP-S) (3) and 4-ethylphenol (ETP) (4).

The optimized RP-HPLC assay using a mobile phase of methanol-0.01 M citrate buffer pH 6.2 (47:53, v/v) containing 0.03 M tetrabutyl-ammonium-bromide (TBAB) provided baseline separation of PNP, PNP-G, PNP-S and ETP in less than 14 min of chromatographic time (Figure 2). This analysis time was shorter than those of the previously published isocratic methods for determination [9–12]. Using UV detection at λ = 290 nm, the optimized wavelength for simultaneous detection of PNP, PNP-G, PNP-S, ETP, chromatograms of the blank perfusates and those of the control bile samples did not show any interfering peaks.

Reproducibility of retention times and integrated peak areas was determined by analysis of six different concentrations of PNP, PNP-G and PNP-S. Standard solutions were prepared by addition of known concentration of each solute to drug free perfusate. Each concentration was analyzed five times. The percent RSD values found for within-day retention times and integrated peak areas of the standard solutions are shown in Tables 1–3.

Linear response was obtained for the calibration of all three compounds in the target concentration range of 2.5–100 μM (n = 6). The calibration ranges were chosen based on the expected solute concentrations in the bile [7].

The obtained calibration equations (with r^2^ values) were:

$$\begin{array}{l} \text{y}=0.0142\;\text{x}-0.0021\;(1.000)\;\text{for PNP}-\text{G}\\ \text{y}=0.0080\;\text{x}-0.0007\;(1.000)\;\text{for PNP}\\ \text{y}=0.0142\;\text{x}-0.0007\;(0.9999)\;\text{for PNP}-\text{S} \end{array}$$

Precision of both retention times (RSD = 0.42–4.04%) and the integrated peak areas (RSD = 0.57–5.49%) were found to be acceptable.

ETP gave a linear response in the concentration range of 1–5 mM (n = 6). The calibration equation for this range (with r^2^ value) was found to be

y=1.9247 x-0.0917 (0.9994).

Precision of the integrated peak areas (RSD = 0.44–3.71%) and that of the retention times (RSD = 3.71–4.14%) were found to be satisfactory.

For the calibration curves obtained for PNP, PNP-G and PNP-S the percentage relative standard error (%RSD) of the slopes were 2.57%, 2.93% and 3.04%, respectively. Based on the calculation of the root mean square error (RMSE) over the 2.5–25.0 μM (PNP-G and PNP-S) concentration ranges, the instrumental quantification limit (calculated as (10xRMSE)/m, where m is the slope of the calibration curve [13]) for the PNP-G and PNP-S was found to be 2.7 μM and 2.1 μM, respectively.

For determination of day-to-day precision of retention times and integrated peak areas, one set of calibration samples with the same PNP, PNP-G and PNP-S content was prepared and measured on the same working day over five consecutive weeks. The percentage RSD values for retention time and integration areas obtained from the measurements are summarized in Tables 1–3.

**Fig. 3:**
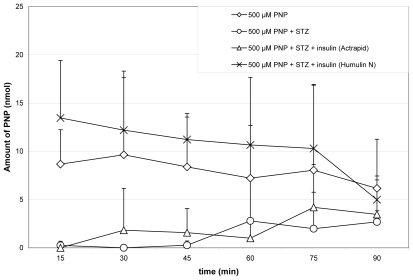
Change in excreted amount of PNP in the bile of the rat during perfusion of the jejunal loop with isotonic medium containing 500 μM PNP. Each value represents the average of eight independent experiments ± standard error. Details of measurements are written in the Experimental part.

**Fig. 4:**
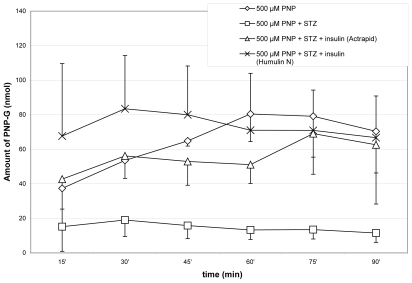
Change in excreted amount of PNP-G in the bile of the rat during perfusion of the jejunal loop with isotonic medium containing 500 μM PNP. Each value represents the average of eight independent experiments ± standard error. Details of measurements are written in the Experimental part.

**Fig. 5:**
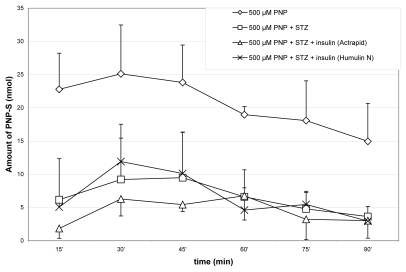
Change in excreted amount of PNP-S in the bile of the rat during perfusion of the jejunal loop with isotonic medium containing 500 μM PNP. Each value represents the average of eight independent experiments ± standard error. Details of measurements are written in the Experimental part.

The method described above has been applied to determination of PNP and the two PNP conjugates in the bile samples of the rat. The concentrations of PNP, PNP-G and PNP-S in the samples were determined by standard curves described above. The graphical representations of the results are shown on Figures 3–5. Each value represents the mean ± standard error (n=8).

## Discussion

In order to set up a simple, easy to perform assay to quantify PNP and its conjugates in the rat bile samples, we considered only isocratic RP-HPLC methods that can provide a straightforward and time-consuming separation of PNP and both of its glucuronide (PNP-G) and sulfate (PNP-S) conjugates. Earlier Karakaya and Carter reported on separation of glucuronide and sulfate conjugates of 4-nitrophenol and 1-naphtol using methanol-water mixtures of different compositions containing 0.01 M TBAB as eluent [14]. The authors found that the two more acidic conjugates (pK_a_ (PNP-S) 4 [15], pK_a_ (PNP-G) 3.0–3.4 [16]) and the weak acid parent PNP (pK_a_ = 7.14 [17]) could be well separated without precise adjustment of the pH of the eluent. Due to the acid-base character and the presence of other components of the bile, however, in the present work we applied a buffer for the precious setting of the pH, and increased the quantity of TBAB, the ion-pairing reagent. To further improve precision of the method, an internal standard (ETP) was applied.

During our preliminary investigations we have performed chromatographic analysis of a mixture of PNP, PNP-G, PNP-S and ETP (100 mM each) using a mobile phase methanol-0.01 M citrate buffer (47:53, v/v) containing 0.03 M TBAB. It was found that the integrated areas remained unchanged when the pH of the mobile phase was 3.5, 4.0, 5.0 and 6.2 but the *k* values of PNP and PNP-S was found to be appropriate for baseline separation only at pH 6.2. Under the optimum pH conditions both PNP-G and PNP-S exist practically fully ionized, which is the appropriate form of the polar species to form ion pairs with TBAB adsorbed onto the RP stationary phase [18].

The method described above has been applied to the determination of PNP and the PNP conjugates (PNP-G and PNP-S) in rat bile samples. The amount of the excreted PNP and its conjugates was determined in control and streptozotocin-treated animals. Streptozotocin (STZ) destroys the insulin producing β-cells in the pancreas, causing a diabetes-like status (hyperglycemia) to the experimental animals [19]. In comparison, determination of the amount of the three compounds has been also performed in bile samples of STZ plus insulin-treated animals. Changes in the excreted amount of PNP, PNP-G and PNP-S in the bile of the rat during perfusion of the jejunal loop with isotonic medium containing 500 μM PNP are demonstrated on Figures 3–5.

As it is shown in Figure 3, the STZ treatment depressed the bile excretion of PNP. The depression was not influenced by the rapid-acting insulin (Actrapid); however, the intermediate-acting insulin (Humulin N) was able to compensate the decrease of biliary excretion of PNP. The STZ treatment decreased excretion to the bile of the PNP-G metabolite as well. The insulin treatments elevated the levels of the metabolite to those of the control (Figure 4).

The experimental diabetes caused by STZ also decreased the PNP-S in the rat bile, and the insulin treatments were not able to compensate this reduction (Figure 5).

In conclusion, the ion pair reversed phase HPLC method with UV detection provided acceptable results in the target quantification range of PNP, PNP-G and PNP-S for within day precision (repeatability), day-to-day precision (reproducibility) and linearity. The method has proved to be simple and easy-to-perform for determination of PNP, PNP-G and PNP-S in the rat bile samples.

## Experimental

### Chemicals

4-Nitrophenol (PNP), 4-nitropheny β-glucuronide (PNP-G), 4-nitrophenyl sulfate (PNP-S), 4-ethylphenol (ETP), streptozotocin (STZ) and tetrabutylammonium bromide (TBAB) were obtained from Sigma-Aldrich (Budapest, Hungary). The structures of PNP, PNP-G, PNP-S and ETP are shown on Figure 1. Actrapid (40 IU/ml rapid-acting insulin) and Humulin N (intermediate-acting insulin) were obtained from Novo Nordisk Hungaria (Budapest, Hungary) and Lilly Hungaria (Budapest, Hungary), respectively. All solvents were HPLC grade.

### HPLC conditions and instrumentation

The HPLC system consisted of a Varian 2010 pump, a Rheodyne 7725i injection valve, a UV-Detector 308 (Labor MIM, Budapest, Hungary) UV-Vis detector. Data were collected and integrated using a PowerChrom 280 data module and software (ADInstruments, Sydney, Australia). A Nucleosil 100 C18 reversed phase column (250 mm × 4.6 mm I.D., 10 μm particle size), and a TR-C-160K1 ODS guard column (Teknokroma, Barcelona, Spain) were employed for all separations. UV measurements were performed on a Secomam Anthelie Light UV-Vis spectrophotometer (Secomam, Ales, France) at ambient temperature. A Mettler Toledo MP 220 pH meter and a Mettler Toledo Inlab 413 electrode (Mettler Toledo, Budapest, Hungary) were used to adjust the pH of the electrolyte solutions.

### Analytical conditions

The mobile phase used consisted of methanol-0.01 M citrate buffer pH 6.2 (47:53, v/v) containing 0.03 M TBAB. The pH of the eluent was adjusted with 1 M HCl solution. The sample volume was 20 μl, the flow rate was 1.0 ml min^−1^ and detection was affected at 290 nm, the wavelength that was found to be the optimum for simultaneous detection of PNP, PNP-S, PNP-G and ETP. The analyses were performed at ambient temperature allowing a 15 min equilibration after the start of pumping mobile phase through the column. The retention factors (*k*) of PNP-G, PNP, PNP-S and ETP were 0.86, 2.51, 3.42 and 4.63, respectively.

### Calibration curves

Standard solutions were prepared by addition of known concentrations (1–100 μM) of each solute (PNP, PNP-G, PNP-S) and ETP (2.5 mM) to the eluent. Calibration curves were constructed based on the ratio of integrated peak areas of the solutes and that of the internal standard. For each solute a linear relationship was found in the 2.5–100 μM concentration range.

### Precision of method

Five replicate injections were made of the standard solutions. The relative standard deviation (RSD) of retention times and peak areas was calculated to assess intra-day precision. The same refrigerated (4 °C) solutions were used to prepare the standard curve for five consecutive days and the RSD values calculated to assess inter-day assay variations.

### Animal experiments

Hyperglycemia was induced in male Wistar rats (weighting 220–250 g) by i.v. administration of STZ in a dose of 65 mg/kg. After 1 week of the STZ treatment animals were anaesthetized with urethane (1.2 g/kg, i.p.). One group of the animals was treated with rapid-acting insulin (Actrapid) (1 IU/kg, i.m.) just before the anesthesia. A second group of the animals was treated with intermediate-acting insulin (Humulin N) (2 ×15 IU, i.p.) every day for one week after the STZ treatment.

The jejunal loop and the bile duct of the anaesthetized rats were cannulated and the lumen of the small intestine was perfused with isotonic medium containing 500 μM PNP at a rate of 13 mL/min in recirculation mode. Samples (250 μL each) were obtained from the perfusion medium coming out from the jejunal loop. Bile was collected in 15 min periods into Eppendorf tubes placed in ice. The rate of the bile flow changed between 37.6±9.95 to 103.1±11.5 (μl/kg/min). The collected samples were stored in refrigerator (−20 °C) until analysis.

All procedures were carried out on animals according to the Hungarian Animals Act (Scientific Procedures, 1998), and the study was approved by the Ethics Committee on Animal Research of the University of Pécs.

### Sample preparation

Before analysis, temperature of the samples was allowed to rise to ambient temperature, and the perfusates were vortexed for 10 s and centrifuged at 3000 g for 10 min. After 10 s vortexing, 50 μl aliquot of each bile sample was taken and mixed with 200 μl of 3.125 mM methanolic ETP solution. The samples were vortex-mixed for 20 s and then centrifuged at 10000 g for 10 min to sediment the precipitated protein. Each measurement represents the average of eight independent experiments.

## Figures and Tables

**Tab. 1:** Within day and day-to day precisions of retention times and peak areas of PNP in standard solution. (The precisions of the peak areas were calculated from the ratios of the peak areas of PNP and ETP)

Concentration of standard solution (μM) PNP	Within-day precision of retention times (RSD, %) (n=5)	Within-day precision of peak areas (RSD, %) (n=5)	Day-to-day precision of retention times (RSD, %) (n=5)	Day-to-day precision of peak areas (RSD, %) (n=5)
2.5	1.15	5.59	3.31	4.77
5	1.05	5.49	3.47	4.31
10	1.40	4.93	3.39	1.31
25	2.13	4.87	3.71	2.26
50	1.72	1.88	3.57	2.83
100	1.01	1.77	4.04	0.86

**Tab. 2:** Within day and day-to day precisions of retention times and peak areas of PNP-G in standard solution. (The precisions of the peak areas were calculated from the ratios of the peak areas of PNP-G and ETP)

Concentration of standard solution (μM) PNP-G	Within-day precision of retention times (RSD, %) (n=5)	Within-day precision of peak areas (RSD, %) (n=5)	Day-to-day precision of retention times (RSD, %) (n=5)	Day-to-day precision of peak areas (RSD, %) (n=5)
2.5	0.50	4.96	1.26	1.30
5	0.50	4.61	1.28	2.44
10	0.45	4.40	1.17	1.34
25	0.42	4.19	1.69	1.64
50	0.51	1.40	1.33	2.02
100	0.57	1.63	1.51	0.79

**Tab. 3:** Within day and day-to day precisions of retention times and peak areas of PNP-S in standard solution. (The precisions of the peak areas were calculated from as the the ratios of the peak areas of PNP-S and ETP)

Concentration of standard solution (μM) PNP-S	Within-day precision of retention times (RSD, %) (n=5)	Within-day precision of peak areas (RSD, %) (n=5)	Day-to-day precision of retention times (RSD, %) (n=5)	Day-to-day precision of peak areas (RSD, %) (n=5)
2.5	0.83	4.21	3.34	2.40
5	0.92	3.30	3.30	2.88
10	1.04	3.42	3.21	2.68
25	1.02	3.50	3.25	2.58
50	1.41	3.36	3.34	2.41
100	1.23	3.48	3.79	0.57
